# Management of fetal death complicated by placenta previa during the midtrimester

**DOI:** 10.1002/ccr3.1012

**Published:** 2017-05-31

**Authors:** Sayuri Nakanishi, Ryosuke Shindo, Shigeru Aoki

**Affiliations:** ^1^ Perinatal Center for Maternity and NeonatalYokohama City University Medical CenterYokohamaJapan

**Keywords:** Expectant management, fetal death, placenta previa, vaginal delivery

## Abstract

Expectant management of fetal death complicated by placenta previa occurring during midtrimester trimester may induce fetal/placental atrophy and decrease uterine blood flow to facilitate vaginal delivery. Our experience with these cases suggests that about 4 weeks of expectant management should be considered as a management strategy.

## Introduction

Although infrequent, fetal death complicated by placenta previa during the midtrimester is a very difficult problem because no consensus has been reached on the appropriate delivery strategy and management. As cesarean delivery for a dead fetus carries significant maternal risks of bleeding and adversely affects the next pregnancy without any benefit for the fetus, it should be avoided to the extent possible.

On the other hand, management of midtrimester fetal death in the presence of placenta previa is a dilemma for obstetricians because induction of labor could cause massive bleeding from abruption of the placenta covering the internal cervical os.

We report two cases of fetal death complicated by placenta previa in which the fetuses were delivered vaginally without excessive bleeding after the onset of spontaneous labor following expectant management to allow fetal/placental atrophy.

## Case Examination

**Figure 1:**
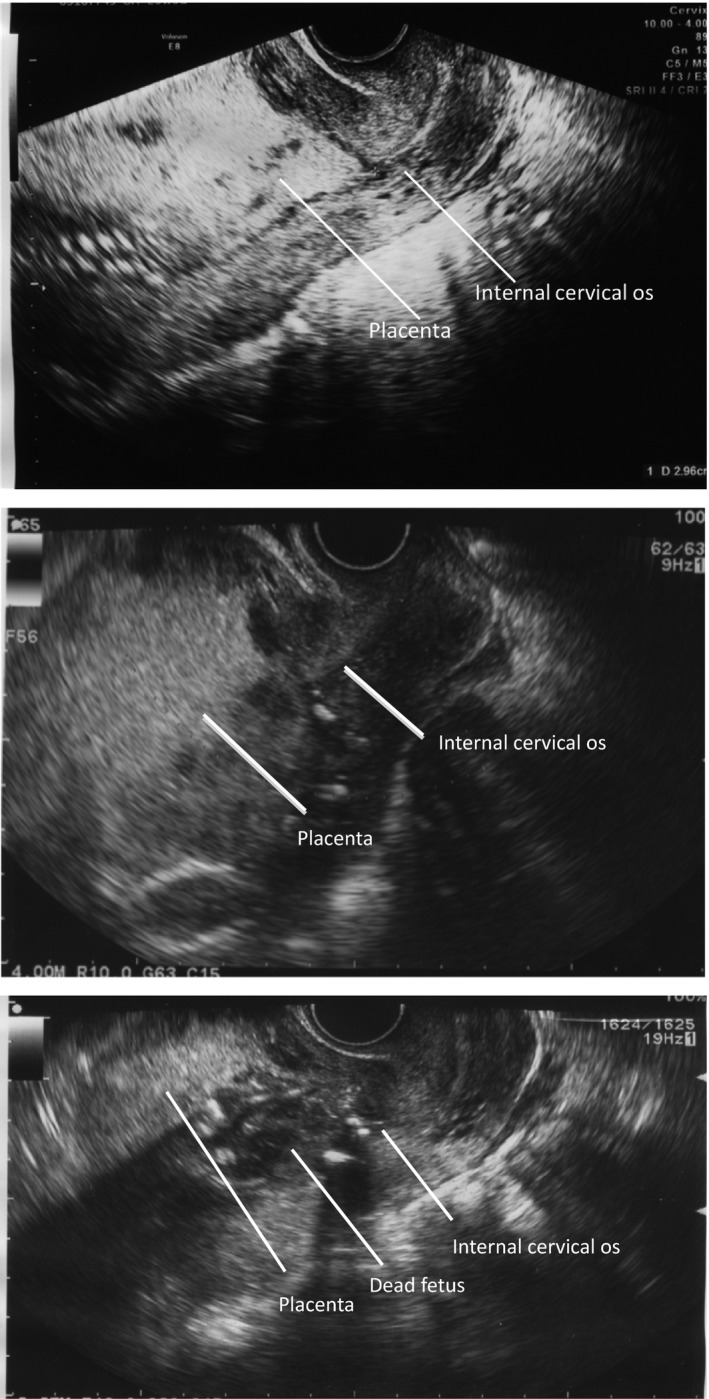
Case 1. Ultrasonographic image taken at 25 weeks and 1 day of gestation. Placenta previa: the placenta completely covers the internal cervical os. Ultrasonographic image taken at 26 weeks and 0 day of gestation. The placenta completely covers the internal cervical os. Ultrasonographic image taken as 28 weeks and 6 days of gestation (22 days after intrauterine fetal death). The placenta is atrophic and no longer covers the internal cervical os.

Case 1 was a 33‐year‐old primiparous woman. The patient was referred to our center with severe fetal growth restriction (estimated fetal body weight, 177 g) and placenta previa (Fig. 1) at 25 weeks and 1 day of gestation. At 25 weeks and 5 days of gestation, fetal death was diagnosed based on the absence of the fetal heartbeat during the follow‐up ultrasonography examination. Although dilation and evacuation (D&E) or induction of labor with a gemeprost vaginal suppository was considered, we chose expectant management, anticipating possible massive bleeding due to mechanical dilation of the uterine cervix, placental atrophy after fetal death, the potential misdiagnosis of placenta previa due to an undeveloped lower uterine segment, and rare consumptive coagulopathy associated with the prolonged retention of a dead fetus. At 28 weeks and 6 days of gestation (22 days after intrauterine fetal death was detected), ultrasonography (USG) showed placental atrophy, resulting in the internal cervical os no longer being covered. (Fig. 1) Blood tests performed during the expectant management did not indicate infection or coagulation disorder.

At 29 weeks and 4 days of gestation (27 days after intrauterine fetal death was detected), labor began and the stillborn fetus was delivered vaginally uneventfully. The duration of delivery was 56 minutes. The blood loss was 383 mL. The fetus weighed 102 g, and the placenta weighed 96 g. The fetus had a severely macerated appearance. The mother made an uneventful postpartum recovery and was discharged the next day.

**Figure 2:**
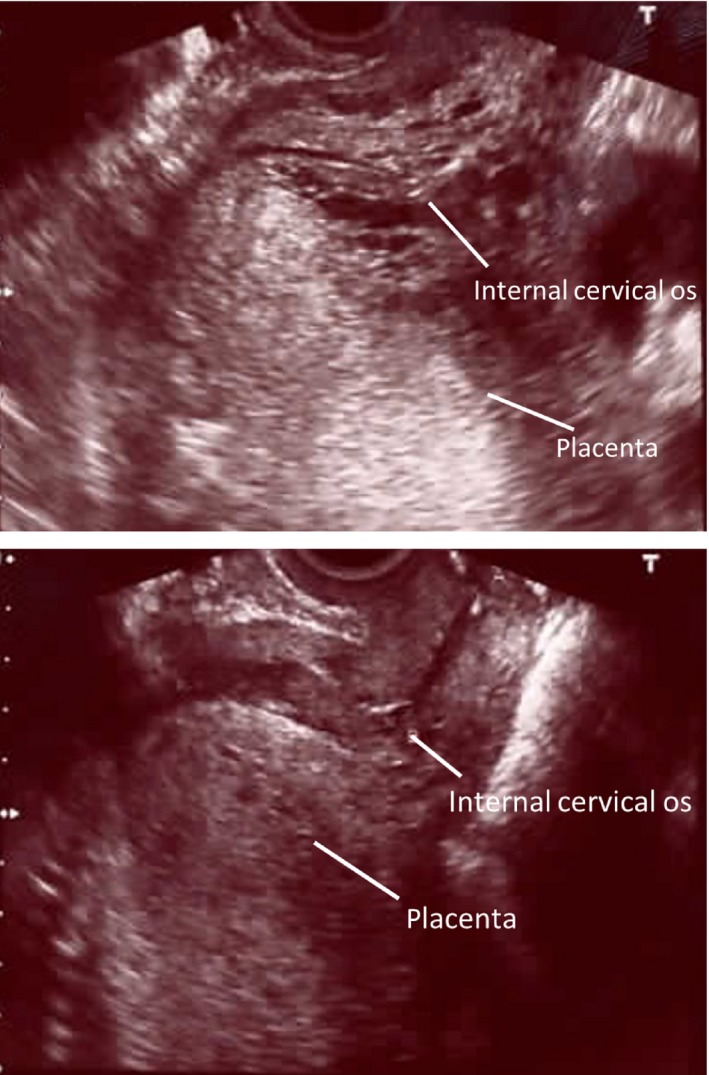
Case 2. Ultrasonographic image taken at 27 weeks and 3 days of gestation. Placenta previa: the placenta completely covers the internal cervical os. Ultrasonographic image taken at 30 weeks and 2 days of gestation. The placental margin partially covers the internal cervical os.

Case 2 was a 42‐year‐old primiparous woman. Placenta previa was noted at 18 weeks and 0 days of gestation. The patient's pregnancy course was uneventful with no episodes of bleeding; however, fetal death was confirmed at 27 weeks and 3 days of gestation when no fetal heartbeat was detected during a prenatal examination. USG found the placenta completely covering the internal cervical os.(Fig. 2) The estimated fetal weight was 790 g. As in Case 1, this case of fetal death complicated by placenta previa was placed under expectant management.

At 29 weeks and 5 days of gestation (16 days after intrauterine fetal death was detected), placental atrophy was noted, and the placental margin was in contact with part of the internal cervical os.(Fig. 2) Blood tests performed during the expectant management did not show any abnormalities. At 30 weeks and 3 days of gestation (21 days after intrauterine fetal death was detected), labor began. After the onset of labor, cervical dilation reached 4 cm and a protruding amniotic sac was identified on speculum examination. However, the placenta was not felt during an internal examination. Labor progressed uneventfully to vaginal delivery. The duration of labor was 5 h and 33 min. The blood loss was 211 mL. The fetus weighed 617 g, and the placenta weighed 200 g. The stillborn fetus had a severely macerated appearance. The mother made an uneventful postpartum recovery and was discharged the next day.

## Discussion

We report two cases of fetal death complicated by placenta previa in which the stillborn fetuses were delivered vaginally without artificial intervention following an expectant management strategy. Two advantages of an expectant management strategy for cases of fetal death complicated by placenta previa are as follows.

First, reduced total blood loss can be expected during delivery because the placenta will have become atrophic over time, and uteroplacental blood perfusion will decrease after stillbirth. Moreover, the placenta previa may become resolved due to placental atrophy. Second, most women will deliver spontaneously within 4 weeks of fetal death.

The lower edge of the placenta in these cases of placenta previa became relocated away from the internal cervical os during expectant management, and the fetus was vaginally delivered without massive bleeding in both cases. Although fetal death complicated by placenta previa has been reported in several studies 1, 2, 3, no large‐scale study on the management of fetal death with placenta previa has yet been reported. Ploeg et al. 4. compared three cases of fetal death with placenta previa occurring at or after 20 weeks of gestation and that were treated with expectant management, induction of labor, and selective cesarean section, respectively. They reported that delivery following expectant management was the safest option with the least amount of blood loss because of fetal and placental atrophy. Ruano et al. 5. performed therapeutic pregnancy termination in 15 patients with placenta previa at 18–31 weeks of gestation. Blood loss was significantly lower in six patients who had an expectant management period of 2–14 days after feticide compared with nine patients who did not receive feticide. While four of nine patients who did not undergo feticide required blood transfusion, none of the patients who underwent feticide did. The study concluded that feticide and the following expectant management period reduced blood loss after the induction of labor. These reports support the rationality of expectant management following fetal death complicated by placenta previa for reducing blood loss during delivery.

On the other hand, Taki et al. 2. reported blood loss of 1900 mL in a case of fetal death that occurred at 23 weeks of gestation in the presence of placenta previa, for which labor was induced with gemeprost after mechanical cervical ripening at 3 weeks of expectant management. Mechanical cervical ripening may have triggered the excessive bleeding in this case. However, the rates of accurate diagnosis of placenta previa at 20–23 weeks of gestation and at 28–31 weeks of gestation are low (49% and 52%, respectively) 6. The diagnosis of placenta previa is not easy when the lower uterine segment is not extended. Although Taki et al. 2. reported that transvaginal ultrasound performed prior to delivery found the placenta located at the internal os, the placenta was not identified at the time of delivery in the present two cases. Therefore, these cases may have had a low‐lying placenta rather than placenta previa.

Because spontaneous labor occurred within 4 weeks of fetal death, the fetus was vaginally delivered in both cases without consumptive coagulopathy associated with prolonged retention of a dead fetus (dead fetus syndrome). If the dead fetus is undelivered, 75% of women enter spontaneous labor within 2 weeks, and about 90% do so within 3 weeks 7. All reports on the time from fetal death to delivery date back to a time when fetal death was difficult to diagnose. In our patients, the fetuses were delivered 27 days and 21 days after intrauterine fetal death. Although it may take longer for labor to begin after fetal death, most patients go into spontaneous labor within 4 weeks. Once spontaneous labor starts, vaginal delivery will be possible without artificial intervention. Expectant management, however, may have disadvantages. In addition to mental stress on the mother who continues to carry a dead fetus, consumptive coagulopathy associated with prolonged retention of a dead fetus (dead fetus syndrome) may occur. However, gross disruption of maternal coagulation rarely develops before 4 weeks 8. According to a survey conducted by Pritchard et al. 9. in over 100 women who had experienced fetal death at least 1 week earlier, a decrease in fibrinogen levels to <150 mg/dL was not reported in any of the women within 5 weeks after fetal death. Therefore, an expectant management period of about 4 weeks does not appear to pose any health risks in mothers.

We recommend an expectant management period of about 4 weeks in cases of fetal death complicated by placenta previa during midtrimester because the onset of consumptive coagulopathy in the mother within 4 weeks is extremely rare, substantial fetal and placental atrophy can be expected during the 4‐week expectant management period, and the onset of spontaneous labor during that period is highly likely. If labor does not begin after 4 weeks, D&E or induction of labor should be considered. Cesarean delivery should be the last option.

In summary, about 4 weeks of expectant management should be considered as a management strategy when fetal death occurs with fetal death complicated by placenta previa during the midtrimester.

## Authorship

SN: contributed to the study design and finalization of the manuscript. RS: wrote the first draft of the manuscript. SA: provided the study design and supervised the study.

## Conflict of Interest

None declared.
